# A parasitic nematode induces dysbiosis in susceptible but not resistant gastropod hosts

**DOI:** 10.1002/mbo3.1346

**Published:** 2023-03-10

**Authors:** Laura Sheehy, Kerry MacDonald‐Howard, Chris D. Williams, Gareth D. Weedall, Hayley Jones, Robbie Rae

**Affiliations:** ^1^ School of Biological and Environmental Sciences Liverpool John Moores University Liverpool UK; ^2^ Royal Horticultural Society Garden Wisley, Woking Surrey UK

**Keywords:** biocontrol, gastropods, metagenomics, microbiome, nematodes

## Abstract

Animals’ gut microbiomes affect a wide array of biological processes including immunity and protection from pathogens. However, how the microbiome changes due to infection by parasites is still largely unknown, as is how the microbiome changes in hosts that differ in their susceptibility to parasites. To investigate this, we exposed two slug species of differing susceptibility to the parasitic nematode *Phasmarhabditis hermaphrodita* (*Deroceras reticulatum* is highly susceptible and *Ambigolimax valentianus* resistant to the nematode) and profiled the gut microbiota after 7 and 14 days. Before infection, both slug species’ microbiota was dominated by similar bacterial genera: *Pseudomonas* (by far the most abundant), *Sphingobacterium, Pedobacter, Chryseobacterium*, and *Flavobacterium*. In the resistant host *A. valentianus*, there was no significant change in the bacterial genera after infection, but in *D. reticulatum*, the bacterial profile changed, with a decrease in the abundance of Pseudomonadaceae and an increase in the abundance of Flavobacteriaceae and Sphingobacteriaceae after 7 days postinfection. This suggests nematode infection causes dysbiosis in hosts that are susceptible to infection, but the microbiome of resistant species remains unaltered. In summary, the regulation of the immune system is tightly linked with host survival, and nematode infection can alter the microbiome structure.

## Graphical abstract

What role does the microbiome play when we are infected with parasites? Using 16S metagenomics, we found one slug species infected with parasitic nematodes undergoes dysbiosis, however, a resistant slug does not. The nematode could be manipulating the host microbiome, or the bacterial communities may be altered due to ill health. How the resistant slug combats nematodes is unknown, but perhaps the bacterial communities in the microbiome play a role.

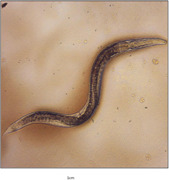

## 1 INTRODUCTION

Bacteria are ubiquitous across the world, often sharing habitats with multicellular life, even colonizing animals either as pathogens or as symbionts (McFall‐Ngai et al., 2013). Developments in next‐generation sequencing have enabled the widespread application of genomics to investigate animal hosts and their bacterial associations, revolutionizing the understanding of these interactions at the level of the microbiome (Bahrndorff et al., 2016). It is well‐established that the microbiome plays an important role in the health of the animal (Fan and Pedersen, 2021; Peixoto et al., 2021; Wu and Wu, 2012). The immune system of the animal host continuously interacts with the microbiome, it is known that the microbiome plays a crucial role in the development of a host's immune system (Zheng et al., 2020).

A host and its microbiome have complex interactions at many levels often undergoing co‐evolutionary pressures, as such it is not correct to truly consider them separate entities. Instead, it is preferable to consider the host and its microbial community as a holobiont recognizing its diversity and dynamic association (Theis et al., 2016). Brealey et al. (2022) suggested that parasites can also be considered holobionts, as they are residing within the microbial community of their holobiont host. Brealey et al. demonstrated that infection by the intestinal cestode *Eubothrium* spp. is associated with dysbiosis of the Atlantic salmon gut microbiome. The cestode *Eubothrium* spp. was shown to select for bacteria belonging to the family mycoplasmas when infecting Atlantic salmon, this highlights the importance of considering the parasite holobiont when studying parasitic infections (Brealey et al., 2022).

Currently, most research is focused on vertebrate holobiont systems, yet invertebrate systems have the potential to become tractable laboratory models for holobiont research due to their relatively simple gut communities and ease of laboratory culture (Newton et al., 2013). Cardoso et al. (2012), Landry et al. (2015), and Jackson et al. (2021) investigated the effects of environment and diet on invertebrate microbiomes showing how the microbiome can shift due to external factors. Cardoso et al. (2012) demonstrated that the microbiome of the land snail *Achatina fulica* can be altered by their diet, with a high‐sugar diet leading to an increase in the abundance of phyla Bacteroidota and Bacillota. Landry et al. (2015) discovered that the majority of bacteria found in the spruce budworm (*Chroistoneura fumiferana*) microbiome belonged to the phylum Pseudomonadota, and their experiments showed species diversity was significantly affected by environment and diet. Jackson et al. (2021) identified and determined that the core microbiome of the slug *Ambigolimax valentianus* can be influenced by diet (sterile and non‐sterile) and environment (garden or lab‐reared). It has also been shown that parasitic infections can alter the balance of the host's microbiome, causing dysbiosis (Brealey et al., 2022) but more research is needed to fully understand this effect. Furthermore, while many studies have concentrated on freshwater parasites and hosts there is little information about the role the gut microbiome plays in host immunity in terrestrial environments.

**Figure 1:**
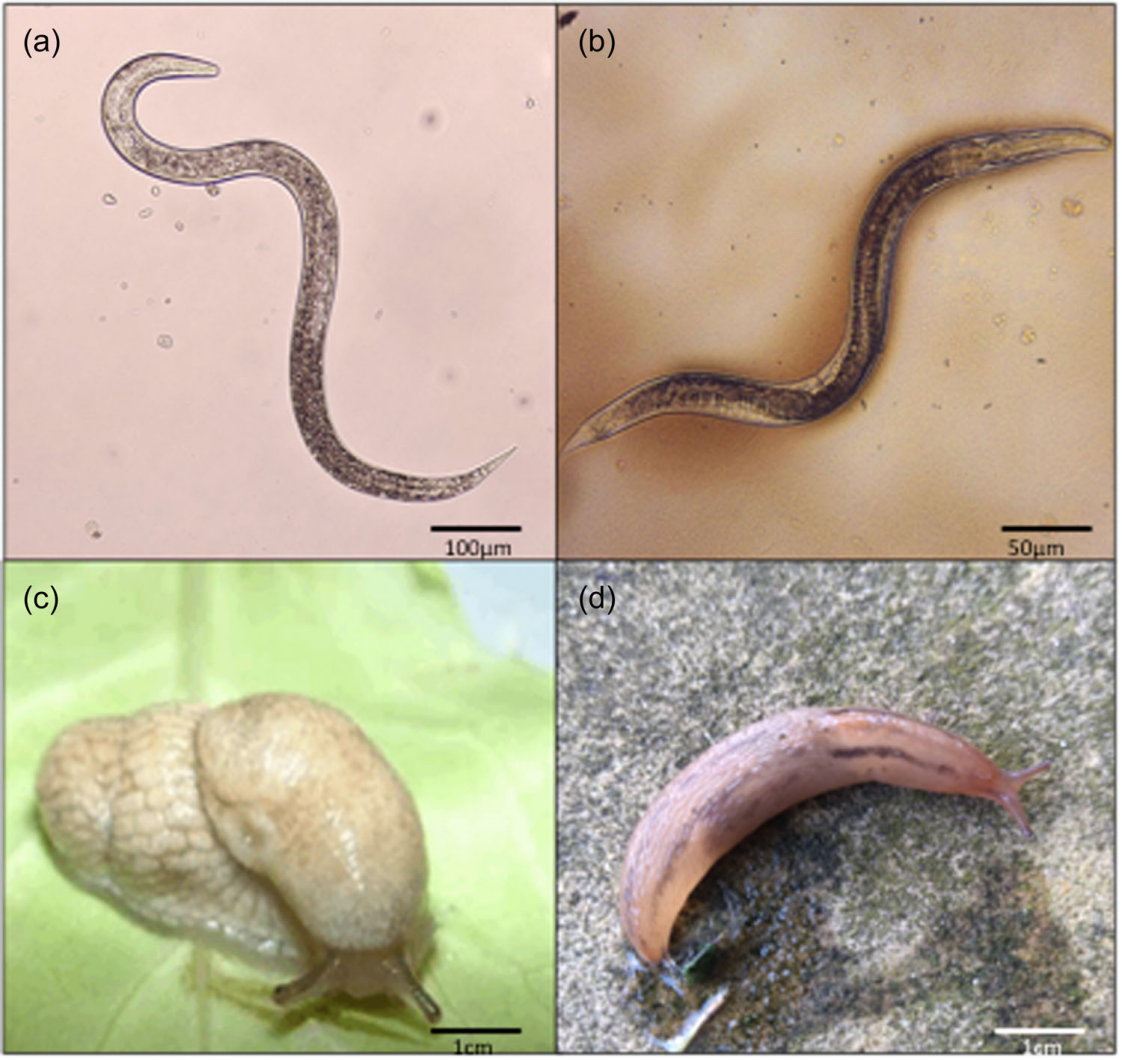
The infective juvenile stage of *Phasmarhabditis hermaphrodita* (a) develops into self‐fertilizing adults (b) that reproduce on a slug cadaver. *P. hermaphrodita* can infect and kill the susceptible slug species *Deroceras reticulatum* (c) but not the resistant slug species *Ambigolimax valentianus* (d).

One such host/parasite system that could provide insight is that of slugs and their nematode parasites. Several slug species are global pests of crops, responsible for millions of pounds of damage each year (Nicholls, 2014). Slugs are parasitized by flies, trematodes, viruses, and microsporidia, but the most prevalent group is the nematodes (Wilson et al., 2004), with 108 species using gastropods as intermediate, definitive, and paratenic hosts (P. S. Grewal, Grewal, Tan, et al., 2003). One species, *Phasmarhabditis hermaphrodita*, is a lethal parasite that has been developed as a biological control agent (Nemaslug®) to control pestiferous slugs on gardens and farms in the UK and northern Europe by BASF Agricultural Specialities (R. Rae et al., 2007). Infective stage nematodes (Figure 1a) are applied to soil and are attracted to chemical cues such as slug mucus and feces (R. G. Rae et al., 2009; Wilson et al., 2006), they then enter through the back of the mantle proliferate, and kill the slug in 4–21 days (Tan and Grewal, 2001b; Wilson et al., 1993). Self‐fertilizing adult nematodes (Figure 1b) reproduce on the cadaver of the slug, and once the food supply is depleted, they develop into new infective juveniles where they search for new slug hosts in the soil. *P. hermaphrodita* has successfully been used to control slugs in many crops including lettuce (Wilson et al., 1995) and in floriculture, for example, orchids (S. K. Grewal, Grewal, Hammond, et al., 2003). *P. hermaphrodita* is not the only nematode to kill slugs, there are several other species, for example, *P. californica* and *P. neopapillosa* that can kill susceptible slugs (Sheehy et al., 2022). There is natural variation in the pathogenic ability of *P. hermaphrodita* (Cutler and Rae, 2020) and crucially, *P. hermaphrodita* (and other *Phasmarhabditis* species) are facultative parasites that can be cultured under laboratory conditions allowing infection experiments to be carried out (see Cutler and Rae, 2020).

*P. hermaphrodita* has been shown to infect and kill several species of pestiferous slug (Rae et al., 2009; Wilson et al., 1993) though the mechanism of pathogenesis is debated. It was thought the nematodes vectored the bacterium *Moraxella osloensis* into the hemocoel of the slug, and this was responsible for host death (Tan and Grewal 2001a, 2001b, 2002). However, recent research by Sheehy et al. (2022) failed to find this bacterium in the next generation of *P. hermaphrodita* (and two other *Phasmarhabditis* species, *P. californica*, and *P. neopapillosa*) after killing a slug using 16S rRNA metagenomic sequencing (Sheehy et al., 2022). Furthermore, these authors showed using 16S rRNA amplicon sequencing that *M. osloensis* is a *Psychrobacter* spp. Therefore, the role bacteria play in causing death to slugs is currently unknown. This warrants further research as these are the only genera of the nematode to evolve to kill slugs and snails out of the entire Nematoda phylum (consisting of an estimated one million species).

We aimed to discover whether *P. hermaphrodita* affects the gut microbiota of two gastropod host species of differing susceptibility to the nematode. The highly susceptible slug *Deroceras reticulatum* (Figure 1c), a common pest species with worldwide importance (Hutchinson et al., 2014), is killed by *P. hermaphrodita* in 4–21 days (Rae et al., 2009; Wilson et al., 1993) while *Ambigolimax valentianus* (Figure 1d) is resistant to being killed by *P. hermaphrodita* (Dankowska, 2006; Ester et al., 2003). How and why these slug species differ so dramatically in their susceptibility to *P. hermaphrodita* is unknown. To investigate the potential role of the gut microbiome we used 16S SSU ribosomal DNA metagenomic profiling to track changes in the gut microbiome of each species before and after infection with *P. hermaphrodita*.

## 2 MATERIALS AND METHODS

### 2.1 Source of invertebrates

*P. hermaphrodita* (strain DMG0001) (Nemaslug®) (Figure 1a,b) was purchased from BASF Agricultural Specialities and stored at 4°C for 1 week before the experiment.

*D. reticulatum* (Figure 1c) and *A. valentianus* (Figure 1d) were collected from Maghull, Liverpool (OS grid reference SD373027) and kept in non‐airtight plastic boxes (35 × 23 × 22 cm) lined with moist tissue paper at 15°C for 7 days and checked daily for any signs of infection by *Phasmarhabditis* nematodes, for example, swollen mantle, lesions. The slugs were immediately fed a diet of iceberg lettuce and carrots using protocols by McDonald‐Howard et al. (2022).

### 2.2 Infection of gastropod hosts with *P. hermaphrodita*

We used a standard bioassay to infect *D. reticulatum* and *A. valentianus* with *P. hermaphrodita* (see Cutler and Rae, 2020; Sheehy et al., 2022). Briefly, infective stage *P. hermaphrodita* DMG0001 was added in doses of either 500 or 1000 nematodes in 2 mL of water to cotton bungs at the bottom of separate 20 mL universal tubes. Two adult slugs (either *D. reticulatum* or *A. valentianus*) were added to each tube, a cotton wool plug was placed on top, and the lid was loosely closed. The slugs were exposed for 7 days at 10°C in the dark, after which feces were collected using a pipette tip to transfer to a 2 mL Eppendorf tube for DNA extraction.

### 2.3 Profiling the gut microbiota from feces of *D. reticulatum* and *A. valentianus*

Individuals were grouped as follows. Group 1 (five *D. reticulatum* fed a lab diet for 7 days); Group 2 (five *D. reticulatum* fed a lab diet for 14 days); Group 3 (five *D. reticulatum* fed a lab diet and infected on Day 7 with *P. hermaphrodita* with feces collected 7 days postinfection—14 days in total); Group 4 (three *A. valentianus* fed a lab diet for 7 days); Group 5 (three *A. valentianus* fed a lab diet for 14 days); Group 6 (three *A. valentianus* infected with *P. hermaphrodita* with feces collected 7 days postinfection—14 days in total). Feces were collected from each slug for DNA extraction.

DNA was extracted from feces using DNeasy PowerSoil Pro Kit (Qiagen) following the manufacturer's instructions. The presence of bacterial DNA was checked after extractions using PCR amplification of the hypervariable regions of the 16S rRNA gene. This was carried out using the primers 27f (5′‐AGAGTTTGATCMTGGCTCAG‐3′) and 1492r (5′‐TACGGYTACCTTGTTACGACTT‐3′) (Lane, 1991) with the following thermocycler conditions: 3 min at 95°C followed by 35 cycles of 15 s at 95°C, 30 s at 55°C, 1.5 min at 72°C, and a final step of 8 min at 72°C. Amplicons were visualized using agarose gel electrophoresis to confirm that PCRs had worked; in all cases, bands of the correct size were present, and no amplification of bacterial DNA could be seen in the extraction negative control or the PCR negative control.

DNA samples were sent for 16S rRNA metagenomic sequencing (Novogene). The V4 hypervariable region of the 16S rRNA gene was amplified using the primers 515F (5′‐GTGCCAGCMGCCGCGGTAA‐3′) and 806R (5′‐GGACTACHVGGGTWTCTAAT‐3′). All PCR reactions were carried out with Phusion® High‐Fidelity PCR Master Mix (New England Biolabs). Sequencing libraries were generated with NEBNext® Ultra^TM^ DNA Library Prep Kit for Illumina and quantified via Qubit and Q‐PCR. Libraries were sequenced on an Illumina NovaSeq. 6000 platform to generate 2 × 250 bp paired‐end reads.

Analysis of the raw reads occurred at Novogene using the following method. Paired‐end reads were merged using FLASH (V1.2.7) (Magoč and Salzberg, 2011). Quality filtering on the raw tags was performed under specific filtering conditions to obtain high‐quality clean tags according to the QIIME (V1.7.0) (Caporaso et al., 2010). The tags were compared with the reference database (SILVA database) using the UCHIME algorithm (Edgar et al., 2011) to detect chimera sequences. Detected chimera sequences were then removed to obtain Effective Tags. All Effective Tags were processed by UPARSE software (v7.0.1090) (Edgar, 2013). Sequences with ≥97% similarity were assigned to the same Operational Taxonomic Units (OTUs).

For each OTU, QIIME (Version 1.7.0) in the Mothur method was performed against the SSU rRNA database of SILVA Database for species annotation at each taxonomic rank (Threshold:0.8~1) (Quast et al., 2012). MUSCLE (Version 3.8.31) (Edgar, 2004) was used to obtain the phylogenetic relationship of all OTUs.

OTUs abundance information was normalized using a standard of sequence number corresponding to the sample with the least sequences. OTUs were analyzed for Alpha diversity (Wilcoxon test function) and Beta diversity (AMOVA—Analysis of Molecular Variance) to obtain richness and evenness information in samples. AMOVA was also used to compare the taxonomic compositions of infected and noninfected slugs in weighted PCoA. Analysis of Alpha and Beta diversity were all performed on the normalized data and calculated with QIIME (Version 1.7.0). Significant intragroup variation is detected via MetaStats based on their abundance.

## 3 RESULTS

### 3.1 The microbiomes of *D. reticulatum* and *A. valentianus*

After quality filtering, all groups produced high‐quality data with an average of over 99,000 tags, with 90% of these tags resulting in taxa annotation (Good's coverage of >0.97 for both *D. reticulatum* and *A. valentianus*). The average number of OTUs per sample for each group was similar within species: Group 1: 746, Group 2: 979, and Group 3: 849 for *D. reticulatum* and for *A. valentianus*; Group 4: 452, Group 5: 403, and Group 6: 463. There is a richer diversity of bacteria associating with *D. reticulatum* compared to *A. valentianus* when considering the number of OTUs.

**Figure 2:**
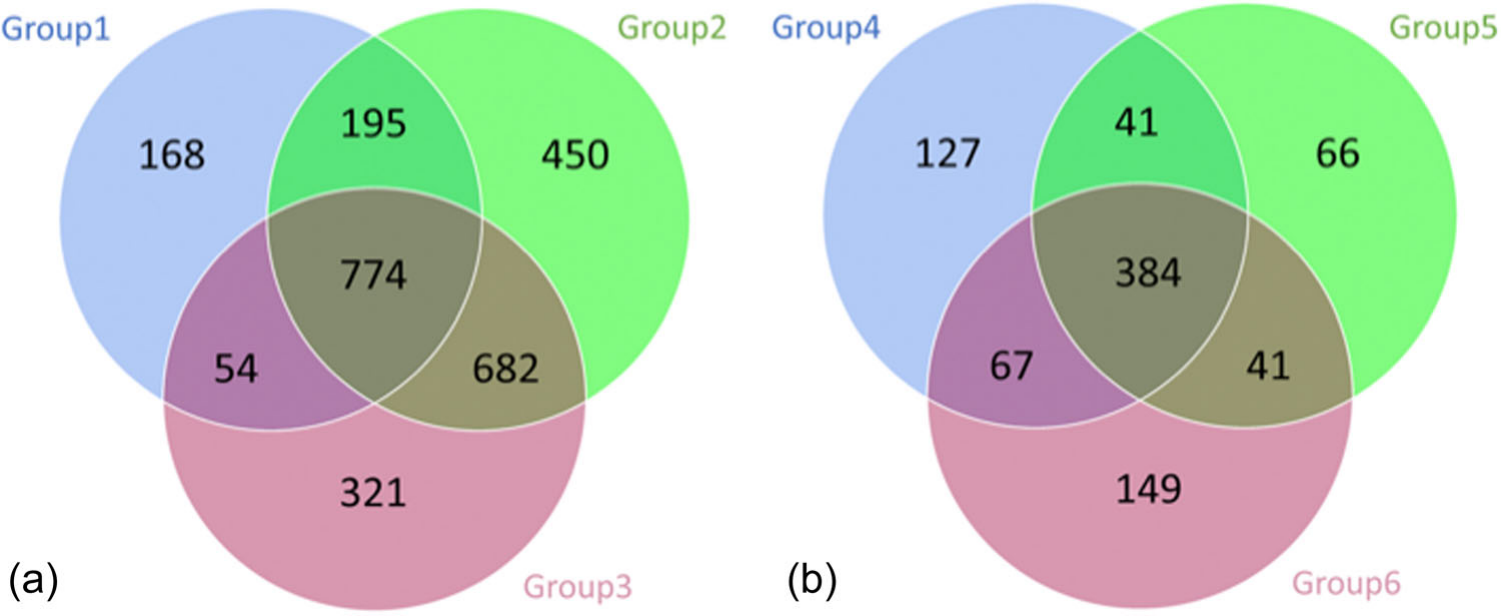
Venn diagrams showing the unique and shared bacterial taxa among groups. (a) *Deroceras reticulatum*. Group 1, fed lab‐diet feces collected on Day 7; Group 2 fed lab‐diet feces collected on Day 14; Group 3, infected with *Phasmarhabditis hermaphrodita* with feces collected 7 days after infection. A large core microbiome of 774 OTUs with 20 phyla represented but dominated by bacteria from the phylum Pseudomonadota. (b) *Ambigolimax valentianus*. Group 4 fed lab‐diet feces collected on Day 7; Group 5, fed lab‐diet feces collected on Day 14; Group 6, infected with *P. hermaphrodita* with feces collected 7 days after infection. A core microbiome of 384 OTUs with just eight phyla represented but in similarity to *D. reticulatum* the core microbiome is dominated by bacteria from the phylum Psuedomonadota.

A core microbiome was identified for *D. reticulatum* with 774 OTUs present (Figure 2a), consisting of bacteria from the phylum Pseudomonadota (39%), Bacteroidota (21%), Bacillota (16%), and Actinomycetota (12%), the remaining 12% spread across 16 phyla (Verrucomicrobiota, Acidobacteriota, Bdellovibrionota, Gemmatimonadota, Chloroflexota, Desulfobacterota, Cyanophyta, Abditibacteriota, Campilobacterota, Methylomirabilota, Patescibacteria, Planctomycetota, Deferribacterota, Nitrospirota, RCP2‐54, and Spirochaetota).

The core microbiome for *A. valentianus* is represented by 384 OTUs across eight phyla (Figure 2b). Bacteria from the phylum Pseudomonadota (54%), Bacterioidota (23%), and Actinomycetota (12%) were the most abundant, with the remaining 11% spread across five phyla (Bacillota, Verrucomicrobiota, Bdellovibrionota, Chloroflexota, and Cyanophyta). While several phyla are represented in both core microbiomes, the abundance of these phyla differs substantially. The core microbiome of *D. reticulatum* shows a much richer diversity than the core microbiome of *A. valentianus*.

**Figure 3:**
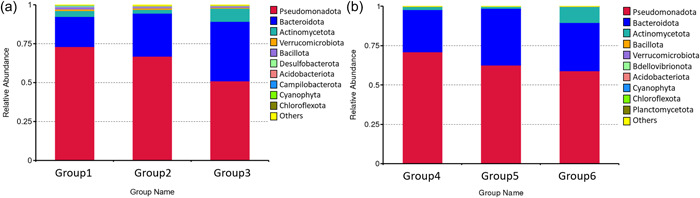
(a) *Deroceras reticulatum* associates with a wide range of bacteria from several phyla Psuedomonadota, Bacillota, Actinomycetota, and Bacteroidota. While the laboratory diet (Group 2) has minimal effect on the relative abundance of these phyla, infection with the malacopathogenic nematode *Phasmarhabditis hermaphrodita* affects the relative abundance of Pseudomonadota and Bacillota. (b) *Ambigolimax valentianus* associates with a wide range of bacteria from several phyla Psuedomonadota, Bacillota, and Actinomycetota. Neither the sustained laboratory diet nor the infection with *P. hermaphrodita* significantly affects the relative abundance of the bacteria associated with *A. valentianus*.

The core microbiomes for *D. reticulatum* and *A. valentianus* indicate several shared bacterial associations at the phylum level. *D. reticulatum* and *A. valentianus* are associated with a wide range of bacteria from several phyla, including Pseudomonadota and Bacteroidota (Figure 3a,b).

### 3.2 No effect of sustained laboratory‐based diet on the microbiome of *D. reticulatum* and *A. valentianus*

The sustained laboratory‐based diet does not lead to a significant difference in the microbiome of *D. reticulatum* or *A. valentianus*. Group 1 (*D. reticulatum*) and Group 4 (*A. valentianus*) were fed a diet of lettuce and carrot for 7 days before feces collection. While Group 2 (*D. reticulatum*) and Group 5 (*A. valentianus*) were fed a diet of lettuce and carrot for 14 days before feces collection. Neither richness (alpha diversity analysis with Wilcoxon test function *p* > 0.5) nor microbiome structure (beta diversity analysis by AMOVA *p* > 0.1) showed a significant change due to the sustained laboratory diet. As shown in Figure 3a,b the relative abundance at the phylum level is similar between Group 1 and Group 2 for *D. reticulatum* and between Group 4 and Group 5 for *A. valentianus*. Therefore, the laboratory‐based diet does not affect microbiome diversity in either slug species.

### 3.3 Malacopathogenic nematode infection results in a microbiome shift in susceptible gastropod hosts but not in resistant host species

**Figure 4:**
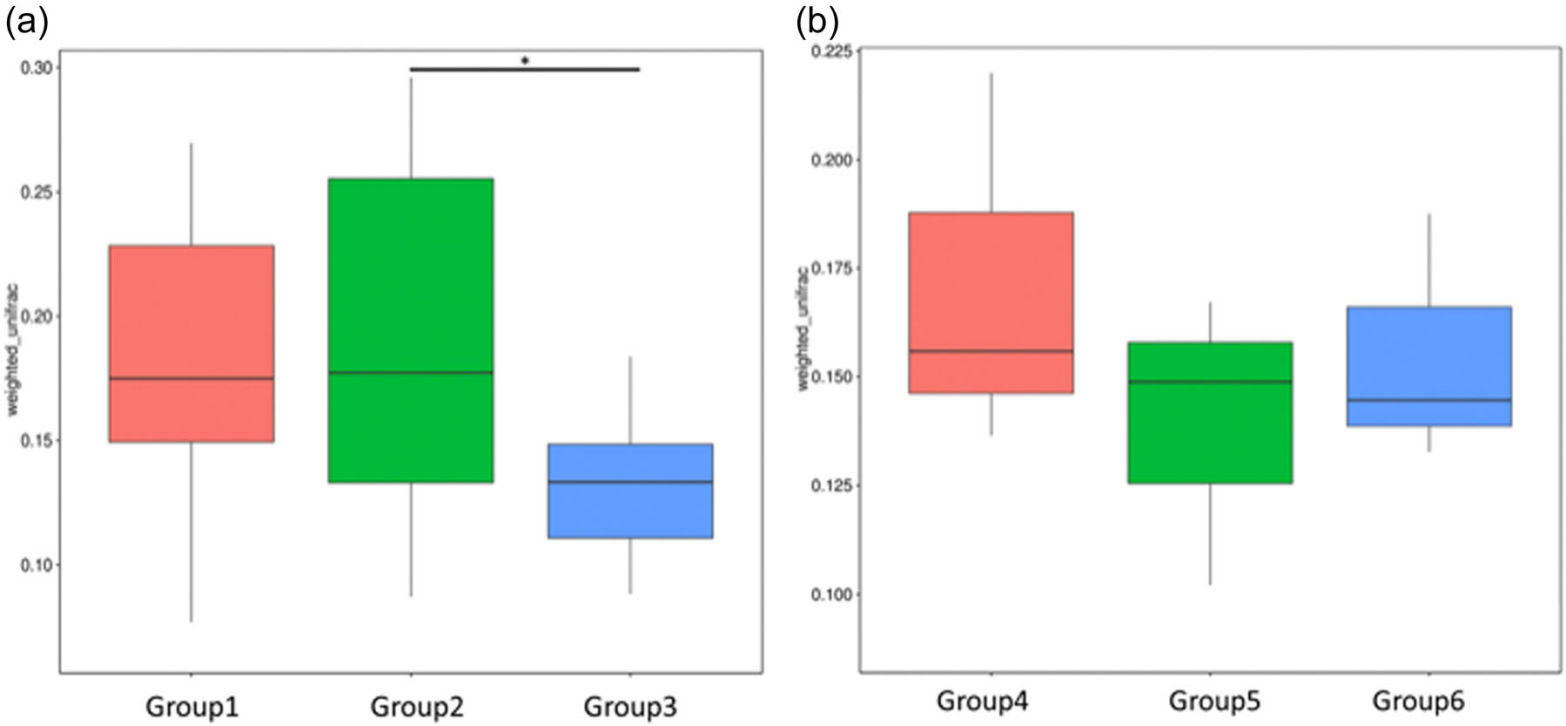
Beta diversity analysis using weighted Unifrac. (a) Wilcoxon test indicates a significant change (*p* < 0.05) in the microbiome structure of the *Deroceras reticulatum* hosts when infected with the malacopathogenic nematode *Phasmarhabditis hermaphrodita*. (b) Wilcoxon test does not indicate a significant change in the microbiome structure of the *Ambigolimax valentianus* hosts when infected with the malacopathogenic nematode *P. hermaphrodita*.

Susceptible *D. reticulatum* hosts infected with *P. hermaphrodita* exhibit a shift in their microbiome (Figure 4a). Beta diversity analysis using the weighted Unifrac Wilcoxon test indicates a significant change (*p* < 0.05) in the microbiome structure of *D. reticulatum* hosts infected with *P. hermaphrodite*. However, resistant *A. valentianus* hosts infected with *P. hermaphrodita* do not exhibit a shift in their microbiome (Figure 4b). Beta diversity analysis using the weighted Unifrac Wilcoxon test indicated no significant change (*p* > 0.05) in the microbiome structure of *A. valentianus* hosts infected with *P. hermaphrodita*.

**Figure 5:**
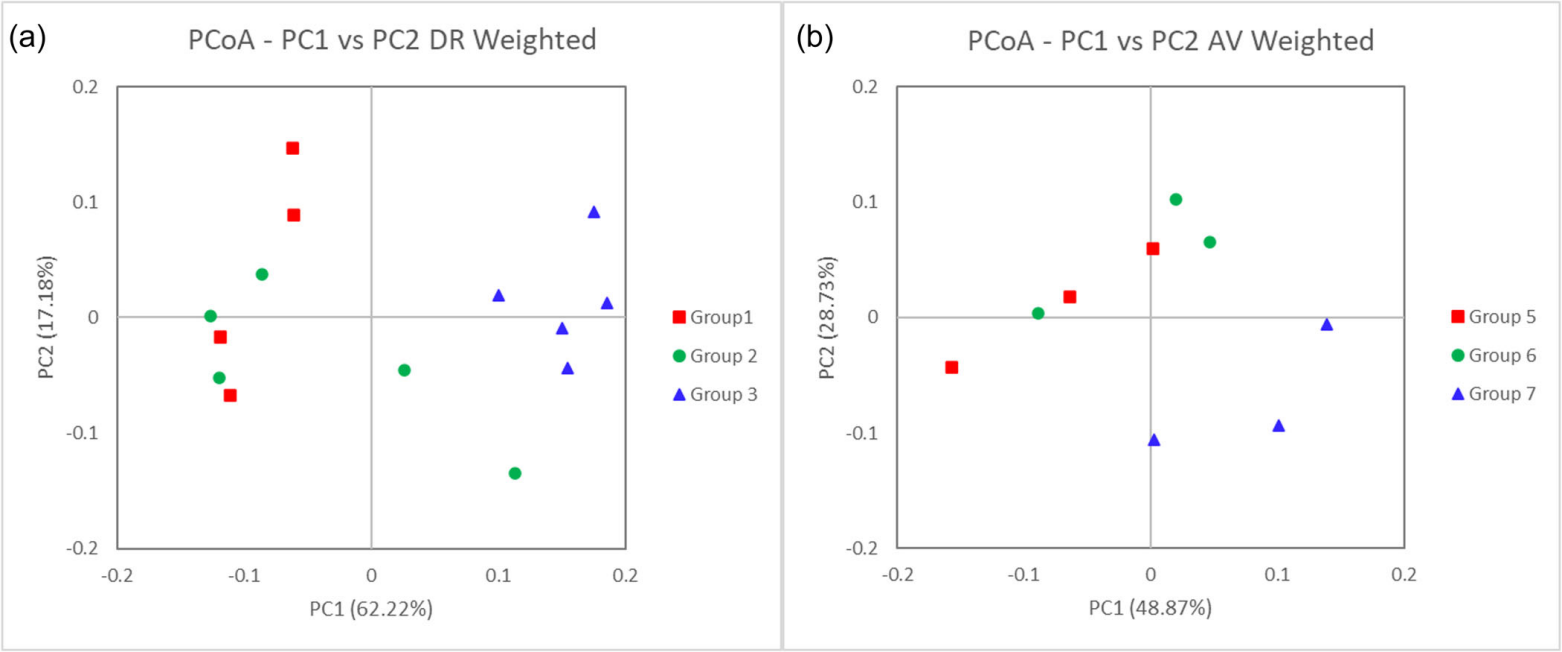
(a) Infection with *Phasmarhabditis hermaphrodita* results in a change in the composition of the microbiome of susceptible slug host *Deroceras reticulatum*. A Principal Coordinates Analysis (PCoA) based on weighted Unifrac distance indicates the taxonomic composition of the communities in each sample. Group 3 shows a significant difference in the composition of bacterial communities than Group 1 or Group 2; this indicates that infection with *P. hermaphrodita* is affecting the microbiome of the host. (b) A PCoA based on weighted Unifrac distance indicates the taxonomic composition of the communities in each sample. Group 6 (infection with *P. hermaphrodita*) results in a slight change in the composition of the microbiome of susceptible slug host *Ambigolimax valentianus*, though this change is not significant.

Infection with *P. hermaphrodita* results in a change in the composition of the microbiome of susceptible slug host *D. reticulatum*. A Principal Coordinates Analysis (PCoA) based on weighted Unifrac distance indicates the taxonomic composition of the communities in each sample (Figure 5). Group 3 shows a significant difference in the composition of bacterial communities compared to Group 1 (*p* < 0.01) and Group 2 (*p* < 0.05) (Figure 5a). This indicates that infection with *P. hermaphrodita* is affecting the microbiome of the host (Figure 5a). PCoA analysis showed there was no significant difference in the composition of the microbiome of *A. valentianus* when infected by *P. hermaphrodita* (Group 6) compared to uninfected *A. valentianus* in Groups 4 and 5 (Figure 5b) (*p* > 0.05).

**Figure 6:**
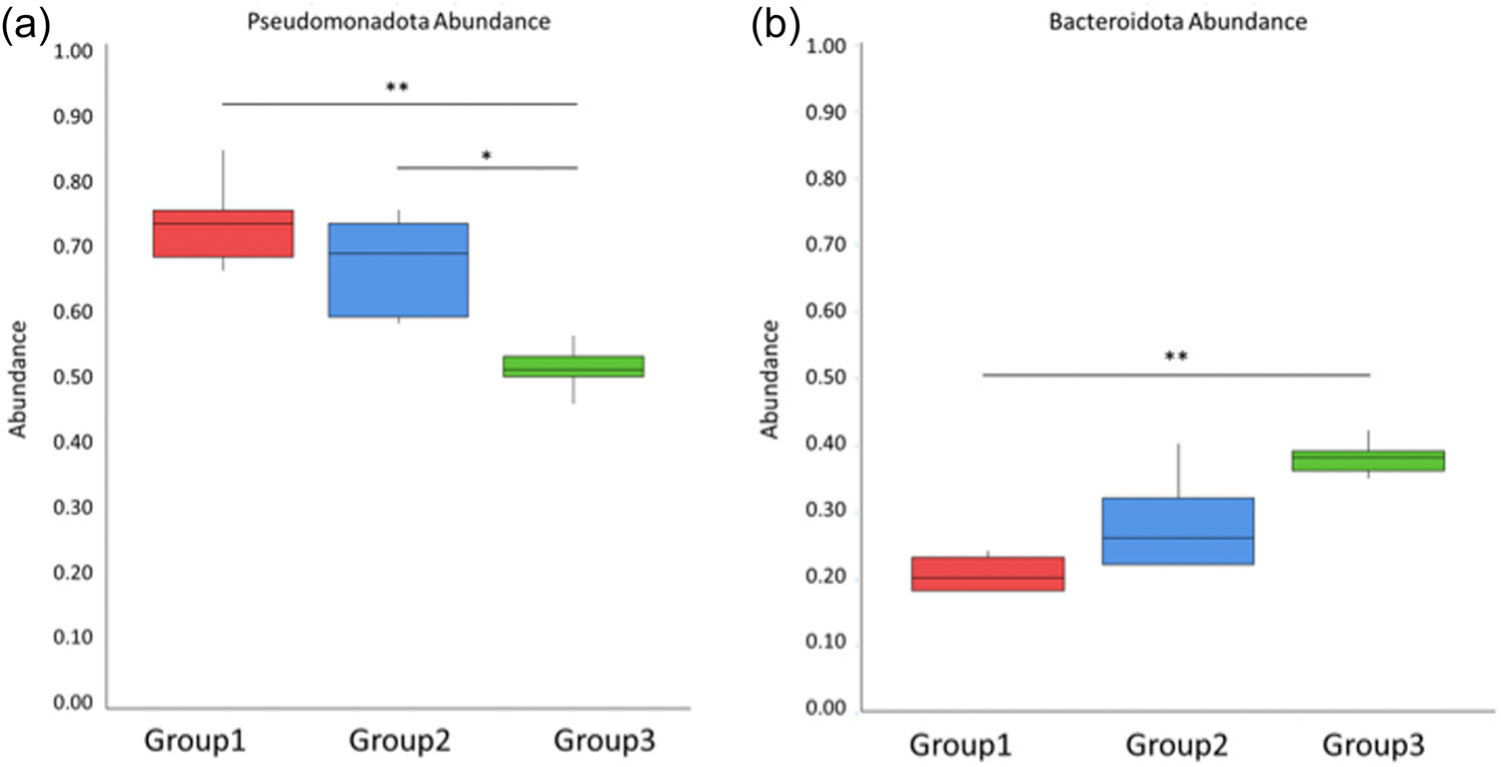
Bacteria with significant intra‐group variation detected via MetaStats based on their abundance. (a) A significant decrease (**p < 0.0005, *p < 0.002) in the abundance of Pseudomonadota in *Deroceras reticulatum* hosts (Group 3) after infection with *Phasmarhabditis hermaphrodita* specifically affected bacteria from the class of Gammaproteobacteria. (b) A significant increase (**p > 0.005) in the abundance of Bacteroidota in *D. reticulatum* hosts (Group 3) after infection with *P. hermaphrodita*.

Bacteria with significant intra‐group variation were detected via MetaStats based on their abundance, through this analysis, a significant decrease in the abundance of Pseudomonadota in *D. reticulatum* hosts was seen after infection with *P. hermaphrodita* between Group 1 and Group 3 (*p* < 0.0005) and Group 2 and Group 3 (*p* < 0.002) (Figure 6a). This decrease specifically affected bacteria from the class of Gammaproteobacteria. Additionally, there are significant decreases (*p* < 0.0005) in the abundance of bacteria in *D. reticulatum* (Group 3) after infection with *P. hermaphrodita* from the following phyla: Thermodesulfobacteriota, Campilobacterota, and Deferribacterota. Conversely, there is a significant increase in the abundance of bacteria from the phylum Bacteroidota in *D. reticulatum* hosts (Group 3) after infection with *P. hermaphrodita* (Figure 6b). Indicating dysbiosis is associated with *P. hermaphrodita* infection.

## 4 DISCUSSION

In this study, we have determined that bacteria from the phyla Pseudomonadota, Bacteroidota, and Actinomycetota were abundantly present in the core microbiomes of *D. reticulatum* and *A. valentianus*. Jackson et al. (2021) also found Pseudomonadota, Bacteroidota, and Actinomycetota to be the most abundant taxa in *A. valentianus* collected in California. These three phyla are commonly found in gastropod microbiomes. For example, Joynson et al. (2017) examined the microbiome of the common black slug, *Arion ater*, and found the most abundant genera to be *Enterobacter, Citrobacter, Pseudomonas, Escherichia, Acinetobacter*, and Sphingobacteriaceae. Reich et al. (2018) found the most abundant phyla in the microbiome of the protected Kerry Spotted Slug *Geomaculus maculosus* to be Pseudomonadota, Bacteroidota, Planctomycetota, Acidobacteriota, Verrucomicrobiota, Bacillota, and Actinomycetota. Both species showed a large abundance of Pseudomonadota before infection, this abundance of Pseudomonadota was also seen in the work from Cardoso et al. (2012) on the microbiome of the giant land snail *Lissachatina fulica*. Though Cardoso also showed that when the land snail was fed a diet rich in sugarcane, there was a greater abundance of Bacteroidota and Bacillota (Cardoso et al., 2012), demonstrating the malleability of the microbiome in response to external forces, in this instance diet.

We also investigated whether and how infection with *P. hermaphrodita* affected the gut microbiota of two host gastropod species. Whilst susceptible host species *D. reticulatum* showed a significant shift in the microbiome, in particular, the abundance of Pseudomonadota and Bacteroidota, the resistant species *A. valentianus* showed no such effect.

Further investigation is still required to understand whether the parasitic nematode directly alters the microbiome of the slug or if the microbiome is shifting as a secondary effect of the ill health of the slug host during a nematode infection. There is evidence in other host/parasite systems to show parasites can directly affect their host's microbiome (Gaulke et al., 2019; Reynolds et al., 2015; Walk et al., 2010). Walk et al. (2010) showed that chronic *Heligmosomoides polygyrus* infection in the duodenum of mice caused an increase in the abundance of Lactobacillaceae and Enterobacteriaceae (Walk et al., 2010). Gaulke et al. (2019) demonstrated that *Pseudocapillaria tomentosa* infection disrupts zebrafish gut microbiome, with a notable increase in abundance of the genus *Mycoplasma* and *Pelomonas* while Fusobacterium and Plesiomonas decreased in their abundance. Through this work, Gaulke et al. (2019) identified interactions between the gut microbiota leading to parasite success.

Furthermore, infection with whipworm (*Trichuris muris*) reduces microbiome alpha diversity in mice (Houlden et al., 2015) but can promote the growth of *Lactobacillus* (Holm et al., 2015). Houlden et al. (2015) also showed that the presence of the parasite is required to maintain the shift in the microbiota as once the parasite is removed the microbiota transitions back to that of an uninfected animal, suggesting that the parasite is directly changing the microbiome of the host rather than a microbiome shift being an indirect response. In addition to the parasites changing the microbiota of the host, White et al. (2018) found the parasitic nematode *T. muris* acquired microbiota from its mouse host, which was needed for its fitness. Interestingly infection by the nematode was only successful if microbiota was present (compared to germ‐free mice). Furthermore, in a comprehensive analysis by Hahn et al. (2022) they showed the microbiome of sticklebacks changes with not just an infection of the cestodes parasite *Schistocephalus solidus*, but this is also dependent on the genotype of the parasite.

It is not only parasitic helminths that can alter the microbiome of their hosts. Koch and Schmid‐Hempel (2011) showed the adaptive value of the microbiota of social bees, which can protect against parasites such as the trypanosomatid gut parasite *Crithidia bombi*. Furthermore, Yun et al. (2022) found strains of honeybees (*Apis cerana*) resistant to sacbrood virus (SBV) had a unique microbiome containing the acetic acid bacterium *Bombella intestine*, the lactic acid bacteria *Lactobacillus* spp. (as well as others) and bees infected with SBV lost crucial bacterial species. The authors suggest a selection of bacteria that could be used as probiotics to keep honeybees healthy. Understanding the multidirectional interactions between parasites, microbiomes, and the host's immune system during infections could open new avenues of treatment and prevention (Reynolds et al., 2015).

Our results show a clear holobiont dysbiosis associated with a *P. hermaphrodita* infection in the susceptible species *D. reticulatum*, but not in the resistant slug species *A. valentianus*, this dysbiosis is consistent with previous studies looking at host/parasite systems in vertebrates (Brealey et al., 2022; McKenney et al., 2015; Walk et al., 2010).

## AUTHOR CONTRIBUTIONS

**Laura Sheehy**: Conceptualization (equal); data curation (equal); formal analysis (equal); investigation (equal); writing—original draft (equal); writing—review & editing (equal). **Kerry MacDonald‐Howard**: Formal analysis (equal); investigation (equal); project administration (equal); writing—review & editing (equal). **Chris D. Williams**: Supervision (equal); writing—review & editing (equal). **Gareth D. Weedall**: Formal analysis (equal); supervision (equal); writing—review & editing (equal). **Hayley Jones**: Supervision (equal). **Robbie Rae**: Conceptualization (equal); supervision (equal); writing—original draft (equal); writing—review & editing (equal).

## CONFLICT OF INTEREST STATEMENT

None declared.

## ETHICS STATEMENT

None required.

## Data Availability

Sequence data are available at the European Nucleotide Archive (ENA) under the ID PRJEB56262 (ERP141185): https://www.ebi.ac.uk/ena/browser/view/PRJEB56262
